# A model system for assessing and comparing the ability of exon microarray and tag sequencing to detect genes specific for malignant B-cells

**DOI:** 10.1186/1471-2164-13-596

**Published:** 2012-11-05

**Authors:** Maria Bro Kloster, Anders Ellern Bilgrau, Maria Rodrigo-Domingo, Kim Steve Bergkvist, Alexander Schmitz, Mads Sønderkær, Julie Støve Bødker, Steffen Falgreen, Mette Nyegaard, Hans Erik Johnsen, Kåre Lehmann Nielsen, Karen Dybkaer, Martin Bøgsted

**Affiliations:** 1 Department of Haematology, Aalborg Hospital, Aarhus University Hospital, Sdr. Skovvej 15, 9000, Aalborg, Denmark; 2 Department of Mathematical Sciences, Aalborg University, Fredrik Bajers Vej 7G, 9220, Aalborg Ø, Denmark; 3 Department of Biotechnology, Chemistry and Environmental Engineering, Aalborg University, SohngårdsholmsvejÂ 49, 9000, Aalborg, Denmark

**Keywords:** Exon microarray, Tag-seq, Gene expression, Detection limit, Sample purity

## Abstract

**Background:**

Malignant cells in tumours of B-cell origin account for 0.1% to 98% of the total cell content, depending on disease entity. Recently, gene expression profiles (GEPs) of B-cell lymphomas based on microarray technologies have contributed significantly to improved sub-classification and diagnostics. However, the varying degrees of malignant B-cell frequencies in analysed samples influence the interpretation of the GEPs. Based on emerging next-generation sequencing technologies (NGS) like tag sequencing (tag-seq) for GEP, it is expected that the detection of mRNA transcripts from malignant B-cells can be supplemented. This study provides a quantitative assessment and comparison of the ability of microarrays and tag-seq to detect mRNA transcripts from malignant B-cells. A model system was established by eight serial dilutions of the malignant B-cell lymphoma cell line, OCI-Ly8, into the embryonic kidney cell line, HEK293, prior to parallel analysis by exon microarrays and tag-seq.

**Results:**

We identified 123 and 117 differentially expressed genes between pure OCI-Ly8 and HEK293 cells by exon microarray and tag-seq, respectively. There were thirty genes in common, and of those, most were B-cell specific. Hierarchical clustering from all dilutions based on the differentially expressed genes showed that neither technology could distinguish between samples with less than 1% malignant B-cells from non-B-cells. A novel statistical concept was developed to assess the ability to detect single genes for both technologies, and used to demonstrate an inverse proportional relationship with the sample purity. Of the 30 common genes, the detection capability of a representative set of three B-cell specific genes - *CD74*, *HLA*-*DRA*, and *BCL6* - was analysed. It was noticed that at least 5%, 13% and 22% sample purity respectively was required for detection of the three genes by exon microarray whereas at least 2%, 4% and 51% percent sample purity of malignant B-cells were required for tag-seq detection.

**Conclusion:**

A sample purity-dependent loss of the ability to detect genes for both technologies was demonstrated. Taq-seq, in comparison to exon microarray, required slightly less malignant B-cells in the samples analysed in order to detect the two most abundantly expressed of the selected genes. The results show that malignant cell frequency is an important variable, with fundamental impact when interpreting GEPs from both technologies.

## Background

Malignant cells in tumour tissues of B-cell origin account for 0.1% to 98% of the total cell content depending on the specific disease entity [1,2]. Varying content in the B-cell tumours of normal leucocytes, lymphocytes and stromal cells with their own individual GEPs poses a challenge for identification of specific malignant B-cell signatures [3]. Although several studies have successfully identified gene expression patterns of tumour samples, their interpretation is often confounded by a lack of information about the varying presence of normal cells within the tumour biopsies. In recent years, microarray technology has been the default technology for detection of global GEP in different cancer types [4]. However, it is difficult to quantify expression of low-abundant mRNA transcripts by microarrays, since low hybridisation intensity signals are difficult to distinguish from background levels arising from non-specific hybridisation. Due to an absence of background signals, NGS technologies, including tag-seq, have the potential to out-perform or at least supplement the exon microarray [5,6]. However, there is a need to explore the technical limits of the exon microarray and tag-seq for identification and interpretation of mRNA transcript profiles, in particular malignant B-cells in a pool of non-malignant cells.

The Affymetrix GeneChip Human Exon 1.0 ST Array is a dense microarray designed for GEP, featuring 6.5 million probes corresponding to known targets and predicted exons spanning the entire human genome [7]. The probes interrogate different regions of the same mRNA transcript showing variation in probe-target hybridisation intensities across the transcript. This variation is sequence-dependent and affected by probe-target binding strength. The probe-target binding strength is affected by competing formation of probe-probe dimers and secondary structures in probes, resulting in background noise from which low hybridisation intensity signals can be difficult to distinguish [5,8]. Gene expression levels are measured as probe-target hybridisation intensities, and all values are background corrected, with the inter-array quantile normalised to remove systematic biases, providing relative rather than absolute gene expression levels [9]. The next-generation sequencing-based tag-seq technology uses two restriction enzymes, *NlaIII* and *MmeI*, to generate cDNA tags that are reverse transcribed from mRNA transcripts, by cutting from the 3′-end CATG to the poly(A)-tail and 17–19 bp downstream of the first restriction enzyme site [6,10,11]. Clonal copies of each cDNA tag are generated on a solid surface flow cell using a bridge amplification approach without any predefined array attached probes before sequencing. During sequencing, the numerical frequencies of each tag are recorded and provide absolute gene expression values [6].

The present study is based on a model system, where malignant B-cells were serially diluted into embryonic kidney cells at eight different cellular frequencies illustrating varying levels of malignant B-cell sample purities. Total RNA from each of the cell populations was tested in parallel to generate data, both by exon microarrays using the Affymetrix GeneChip Human Exon 1.0 ST platform and tag-seq using the Illumina Genome Analyzer platform, as well as RT-qPCR validation of three selected genes. The goal for this work was to develop a quantification method that could be used for assessing and comparing the ability to detect malignant B-cell transcripts based on exon microarray and tag-seq as a function of sample purity.

## Results

### Detection ability of exon microarray and tag-seq

To compare the detection abilities, serial dilutions of eight samples of mixed cell populations from the human malignant B-cell line, OCI-Ly8, into the human embryonic kidney cell line, HEK293, at the cellular frequencies 0%, 0.5%, 1%, 5%, 10%, 20%, 30%, and 100% malignant B-cells, were prepared by fluorescent activated cell sorting (FACS). This dilution scheme was chosen to demonstrate resolution detection for small amounts of malignant B-cells. Total RNA from all samples was prepared and analysed using the two technologies in question. Overall, mRNA expression levels of 17,816 RefSeq transcripts and full-length mRNA transcripts using the core probes of the exon microarray were determined, and the number of unique genes detected above background (DABG) was 11,112. In parallel, exactly the same total RNA from all samples was used to prepare small tags of 17 bp cDNA sequences, which yielded 0.5-2.8 million reads per sample (Table 1). In two of the samples, a high number of reads was noticed. This was probably the consequence of a repetition of the pre-processing for these two samples as they did not meet the quality standards in the first pre-processing. The sub-sampling, however, successfully remedied this problem. Of each sample, 85.4-89.6% of the reads were mapped to the ENSEMBL Homo Sapiens cDNA reference transcriptome (release 63), allowing 1 mismatch per read. Summarising reads from all samples, 134,579 unique reads were identified and 61.5% of these were annotated, while 38.5% did not match any known human mRNA transcript sequence. The unique reads from all eight samples were summarised to gene levels, resulting in the detection of expression of a total of 12,441 unique genes. The average number of tags per gene was 5.8, while 9,784 genes were both DABG by the exon microarray and identified by tag-seq. The entire data generation and analysis flow is illustrated in Additional file 1.

**Table 1:** **Library and annotation results for tag**-**seq data**

**Purity**	**100%**	**30%**	**20%**	**10%**	**5%**	**1%**	**0**.**50%**	**0%**	**Unique**
**Lib**. **size**	543,848	679,415	756,581	2,759,286	805,851	520,166	450,074	2,432,248	134,579
**Annotated**	477,707	580,388	677,669	2,429,976	713,144	459,150	391,854	2,120,204	82,748
**Percent**	87.8	85.4	89.6	88.1	88.5	88.3	87.1	87.2	61.5

### Differentially expressed genes by exon microarray and tag-seq

**Figure 1:**
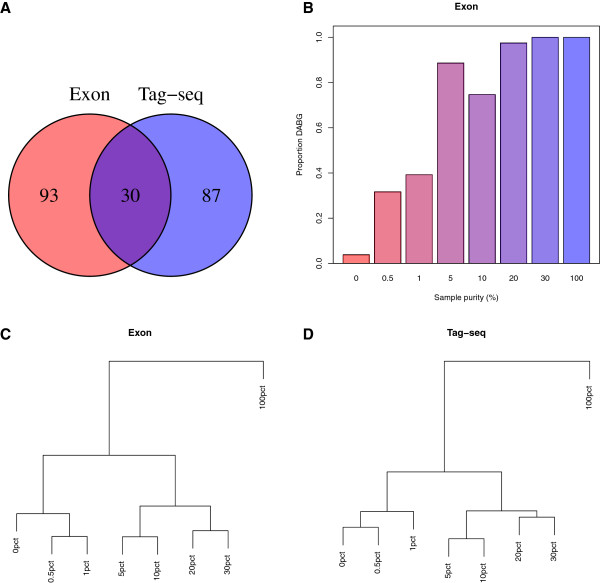
**Characteristics of exon microarray and tag**-**seq data.** (**A**) Venn diagram illustrating numbers of differentially expressed genes identified by exon microarray and tag-seq. An overlap of 30 differentially expressed genes between the pure OCI-Ly8 and HEK293 cell line samples was identified between exon microarray and tag-seq. (**B**) Proportion of B-cell specific mRNA transcripts detected above background for exon microarray. Hierarchical clustering of all cell populations based on differentially expressed genes between the pure samples of OCI-Ly8 and HEK293 cell lines identified by (**C**) exon microarray and (**D**) tag-seq.

Differentially expressed genes were determined between the pure OCI-Ly8 and HEK293 cell line samples using a false discovery rate (FDR) of less than 5%. These settings identified 123 genes by exon microarray and 117 genes by tag-seq. However, only 30 genes were concordant, as indicated in Figure 1A and listed in Table 2. The hypothesis that the common set of genes was caused by pure chance was clearly rejected (*P*-value = 1.07·10^-42^) [12]. A correspondence curve (see Additional file 2) shows that the common set of genes among the most significant genes for each method is not caused by pure chance. Of the 30 concordant genes, 23 genes were highly expressed in the pure OCI-Ly8 cell line compared to the HEK293 cell line. The majority (19/30) of the differentially expressed genes encoded the cluster of differentiation (CD) molecules (*CD20* (*MS4A1*), *CD45* (*PTPRC*), *CD52*, and *CD53*, *CD74*, *CD79A* and *B*), human leukocyte antigen molecules (*HLADRA*), transcriptional regulators (*ELF1*, *ETS1*, *BCL6*, *MYB*, and *EBF1*), B-cell-specific scaffold proteins (*BANK1*), creatine kinase isoenzymes (*CKB*), deubiquitinating enzymes (*UCHL1*), actin-binding proteins (*LCP1*), glycosyltransferases (*ST6GAL1*), uncharacterised proteins (*C13orf18*), and signal transduction molecules (*GCET2*). The full lists of differentially expressed genes are given in Additional files 3 and 4.

**Table 2:** **The 30 overlapping common expressed mRNA transcripts between the OCI**-**Ly8 and HEK293 cell lines identified by exon microarray and tag**-**seq**

**Gene**	**Exon**			**Tag**-**seq**		
	**Log2 FC**	**Adj**. **P**-**val**.	**MDL**	**Log2 FC**	**Adj**. **P**-**val**.	**MDL**
TCL1A	6.976	<0.001	4.5%	10.069	<0.001	1.9%
HLA-DRA	6.135	<0.001	12.6%	9.541	<0.001	4.0%
CD74	5.891	0.001	5.0%	10.077	<0.001	2.0%
C13orf18	6.801	<0.001	6.1%	7.401	0.008	7.9%
ARHGDIB	6.188	<0.001	9.7%	7.349	0.008	15.4%
CKB	−5.573	0.002	NA	−9.526	<0.001	NA
LCP1	7.108	<0.001	7.4%	6.087	0.029	27.4%
CD52	5.63	0.002	9.1%	7.209	0.010	16.9%
FHL1	−5.736	0.001	NA	−6.229	0.028	NA
UCHL1	−5.212	0.006	NA	−7.714	0.005	NA
APBB1IP	5.408	0.003	18.4%	6.267	0.028	25.5%
GLUL	−5.323	0.004	NA	−6.592	0.012	NA
CD79A	6.132	<0.001	4.8%	5.858	0.033	33.1%
GCET2	5.347	0.004	14.8%	6.129	0.029	45.2%
CD79B	4.682	0.020	7.3%	8.077	0.003	7.7%
ELF1	5.07	0.008	6.9%	6.977	0.012	12.7%
CPNE3	−5.036	0.009	NA	−6.304	0.028	NA
CD53	6.122	<0.001	9.0%	5.392	0.049	47.4%
AMOT	−5.189	0.006	NA	−6.044	0.029	NA
ST6GAL1	5.254	0.005	5.8%	5.858	0.028	18.8%
ETS1	5.299	0.005	18.8%	5.781	0.036	26.6%
CNN3	−4.618	0.023	NA	−6.066	0.029	NA
MS4A1	4.435	0.037	13.9%	5.977	0.029	37.9%
MYB	5.117	0.007	14.5%	5.392	0.049	31.8%
APP	−4.419	0.039	NA	−5.858	0.033	NA
PTPRC	4.799	0.015	66.4%	5.392	0.049	67.7%
BANK1	4.677	0.020	35.5%	5.426	0.049	42.6%
ACSL5	4.685	0.020	29.3%	5.392	0.049	64.8%
BCL6	4.366	0.044	21.2%	5.524	0.046	50.2%
EBF1	4.34	0.046	15.1%	5.392	0.049	77.7%

### Hierarchical clustering of all samples

For both exon microarray and tag-seq, hierarchical clustering of all samples based on the 123 and 117 differentially expressed genes resulted in three individual concordant clusters (Figures 1C and 1D). One cluster contained the samples with frequencies of 0%, 0.5% and 1% malignant B-cells, a second cluster of 5%, 10%, 20% and 30% malignant B-cells, and a cluster of the sample containing 100% malignant B-cells. Samples with less than 1% malignant B-cells were not distinguishable by hierarchical clustering from the non-B-cells of HEK293 in either exon microarray or tag-seq. Hierarchical clustering of all samples based on all genes are shown in Additional file 5.

### Comparison of background level and instrument detection limit (IDL)

Of the 123 genes differentially expressed between the 100% and 0% samples detected by exon microarray, we identified 79 B-cell specific genes that were expressed more highly in the 100% B-cell sample. To see whether these 79 B-cell specific mRNA transcripts were present in the background of the non-B-cells, the proportion of B-cell specific mRNA transcripts with DABG was plotted as a function of sample purity (Figure 1B), indicating an increased ability to detect B-cell specific mRNA transcripts as a function of sample purity. Only 4% (3 out of 79) of the B-cell specific mRNA transcripts had DABG for the pure HEK293 cell line sample. The expression values for the 3 transcripts with DABG were 6.21, 7.05, and 7.02 (*HIST1H3I*, *ST6GAL1*, and *ELF*) and therefore only borderline expressed. This indicates that very few false-positive B-cell specific mRNA transcripts were detectable in the pure HEK293 cell line sample. Of the 117 differentially expressed genes between 100% and 0% samples detected by tag-seq, 61 genes were expressed more highly in the 100% B-cell sample. All had counts equal to zero except for 4 genes (*PHYH*, *DNAJC2*, *TCF4* and *ST6GAL1*) that had counts of 2, 1, 3 and 1 respectively in the pure HEK293 cell lines, demonstrating very few, if any, false-positive B-cell specific mRNA transcripts using the tag-seq technology.

**Figure 2:**
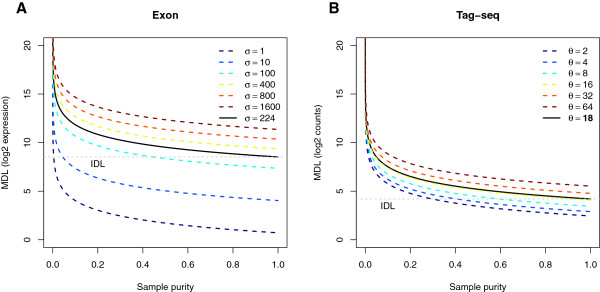
**MDL of exon microarray and tag**-**seq.** MDL plotted as a function of sample purity by (**A**) exon microarray and (**B**) tag-seq. Full drawn black line is the MDL estimate from the experiment. Dotted lines show MDL for a range of standard errors (exon) and dispersion parameters (tag-seq). IDL is the instrument detection limit defined as 367 when 95% confidence was used for exon microarrays and 18 for tag-seq when the linear mean-variance negative binomial regression was used. In the pure sample detection of signal is not possible below the IDL.

To define where true mRNA transcripts were present and where background levels make detection impossible, the instrument detection limits (IDL) of exon microarray and tag-seq were determined. By regression of the mRNA expression levels of the B-cell specific differentially expressed genes versus sample purity under the assumption of a common background as intercept and gene-individual slopes, the IDL was determined for both technologies (Figure 2). For exon microarray, the resulting residual standard deviation was estimated to be *σ* = 224, resulting in an IDL of 367 when 95% confidence was used. The linear model was controlled by standardised residual plots. For exon measurements we noticed 8 outliers and for fitted values below the IDL a clear tendency to measurements being below the regression line (Additional file 6, Figure A). For measurements with a fitted value above the IDL and an absolute value below 3 we noticed a small tendency to increasing variance (Additional file 6, Figure A) and deviation from the normal distribution (Additional file 6, Figure B). For tag-seq, the linear mean-variance negative binomial regression NB1 yielded an IDL of 18, whereas the NB2 model yielded an IDL of 216. The NB1 and NB2 models were controlled by quantile residual plots (see Additional file 6, Figures C, D and E, F, respectively). These plots showed reasonable fits, but the deviance of NB1 (2,556) was lower than the deviance of NB2 (2,763). Therefore we chose to continue with the NB1 model.

### Comparison of method detection limit (MDL)

The ability to detect genes was quantified by plotting the MDL as a function of sample purity (Figure 2). For both exon microarray and tag-seq, the decrease in the ability to detect mRNA expression levels was inversely proportional to sample purity. The MDL met the IDL at 100% sample purity.

**Figure 3:**
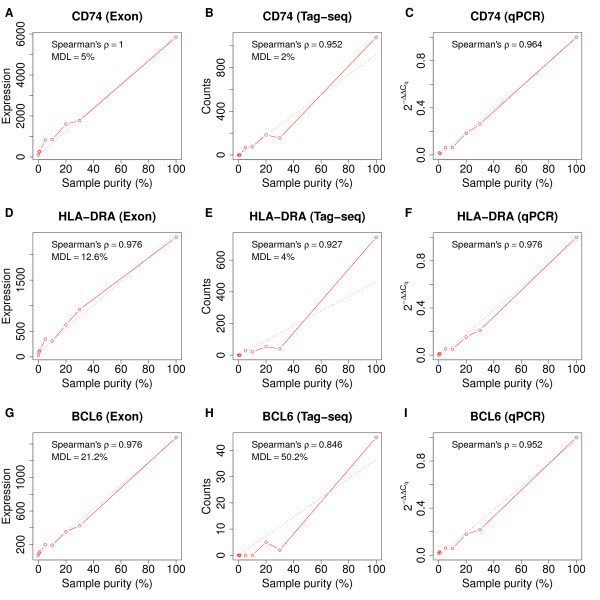
**Detection of B**-**cell specific genes by exon microarray,tag**-**seq,and RT**-**qPCR.** The mRNA expression levels of *CD74*, *HLA*-*DRA* and *BCL6* plotted as a function of sample purity identified by exon microarray (**A**, **D**, **G**), tag-seq (**B**, **E**, **H**), and RT-qPCR (**C**, **F**, **I**).

As *CD74*, *HLA*-*DRA*, and *BCL6* are all transcripts of importance in B-cell development, and they were among the 30 concordant differentially expressed genes between exon microarray and tag-seq, they were selected for comparison and verification of the MDL as a function of sample purity (Figure 3). For exon microarray, the mRNA expression levels of all three genes followed an approximately linear relationship with sample purity (Figure 3A, D, G), whereas larger fluctuations around a linear relationship were observed for all three genes in tag-seq (Figure 3B, E, H). Higher mRNA expression levels were obtained for *CD74* and *HLA*-*DRA* compared to *BCL6* by both exon microarray and tag-seq, supporting that the latter was expressed in low abundance in malignant B-cells. Thus, samples should contain ≥5% malignant B-cells for detection of *CD74*, ≥13% for detection of *HLA*-*DRA* and ≥22% for detection of *BCL6* by exon microarray. Similarly, the tag-seq ability to detect *CD74*, *HLA*-*DRA* and *BCL6* were at least 2%, 4%, and 51% malignant B-cells.

### Validation of gene expression levels by RT-qPCR

The mRNA expression levels of *CD74*, *HLA*-*DRA*, and *BCL6* in all sample dilutions were validated by RT-qPCR and plotted as a function of the sample purity, revealing a linear relationship for all four genes (Figure 3C, F, I). The mRNA expression levels for all three genes, obtained by exon microarray and tag-seq as well as RT-qPCR, were compared by calculating pair wise Spearman’s rank correlation coefficients. High correlations were observed between exon microarray and RT-qPCR, as well as tag-seq and RT-qPCR, for all three genes (Table 3). The exon array showed better correlation to qPCR than tag-seq did.

**Table 3:** **Spearman**’**s rank correlation coefficient of mRNA expression levels between exon microarray**, **tag**-**seq**, **and RT**-**qPCR**

**Gene**	**Exon vs**	**Tag**-**seq vs**	**Exon vs**
	**RT**-**qPCR**	**RT**-**qPCR**	**tag**-**seq**
**CD74**	0.964	0.964	0.927
**HLA**-**DRA**	1.000	0.952	0.952
**BCL6**	0.976	0.873	0.846

## Discussion

The presence of tumour-infiltrating non-malignant cells is expected to mask the detection capacity of GEPs of malignant B-cells [3]. This study, however, suggests a novel quantitative tool for the assessment and comparison of the ability of microarrays and next generation sequencing to detect mRNA transcripts from malignant B-cells in a pool of non-malignant cells. Several studies have compared exon microarrays and next generation technologies [5,13-16]. To the best of our knowledge, no studies have developed quantitative methods which are able to assess the ability of exon microarray and tag-seq to detect transcripts as a function of sample purity. We deliberately chose to make a model system that ensured distinct differences in cellular origin in order to observe clearly differentially expressed genes, enabling us to identify possible difficulties within such a model system.

A comparable number of differentially expressed genes between the pure samples of OCI-Ly8 and HEK293 cell lines was identified by the two technologies (Figure 1A). Although the number of differentially expressed genes in common between the two technologies was small across a number of FDR settings, it was not caused by pure chance (Additional file 2). One factor for this could be false negatives entering due to the lack of replicates at 0% and 100% sample purity. One could also speculate that the small number of commonly expressed genes may be explained by different shortcomings of the platforms, as probes on the exon microarray detect differentially expressed genes that either contain or do not contain the *NlaIII* restriction enzyme site, whereas tag-seq only catches differentially expressed genes with the unique CATG sequence. Therefore, some mRNA transcripts may not be detected by tag-seq due to absence of the CATG sequence, and some mRNA transcripts may not be detected by exon microarrays due to inadequate probe design for exon microarrays [15]. However, we only found 1 gene differentially expressed by exon microarray that did not contain the *NlaIII* restriction site.

A majority of the differentially expressed genes that overlap between exon microarray and tag-seq were B-cell specific mRNA transcripts, including *CD20*, *CD74*, *CD79A*, *HLA*-*DRA*, *BCL6*, *BANK1*, *C13orf18*, and *TCL1A* (Table 2). Most of the B-cell specific genes were related to cell surface-expressed antigens, which is consistent with the importance of interactions with external environment in defining the characteristics of B-cells.

Uniform patterns of relatedness of samples between exon microarray and tag-seq were observed by hierarchical clustering. Even though only 30 of the differentially expressed genes were common between exon microarray and tag-seq, the underlying expression patterns of mRNA transcripts were sufficient to ensure similar results on the relatedness between samples by exon microarray and tag-seq. Samples with ≤1% malignant B-cells were indistinguishable from the pure non-B-cell sample for exon microarray and tag-seq. Thus, >1% malignant B-cells should be presented in biopsies for detection of a malignant B-cell profile by exon microarray and tag-seq, given the model system and data at hand (Figure 1C and 1D).

Based on concepts from analytical chemistry, it was possible to show how the ability to detect single genes (MDL) increases with sample purity (Figure 2). Both exon microarray and tag-seq showed limitations when studying low-abundant mRNA transcripts. A topic for future work is to define the precision estimates of the IDLs, MDLs and background levels as a function of dilution density and replicates. These results are important for designing future detection ability studies. When the precision estimates have been improved with other data sets it will be possible to establish guidelines on how low expression levels of mRNA transcripts are detectable in the original sample for a given sample purity, and thus, give advice on the detection abilities of e.g. low-abundant transcription factors and stem cell genes.

Variance inhomogeneity was observed for the residuals of the linear model used for the exon microarray (Additional file 6, Figure A and B). We noticed that, for fitted values below the IDL, there is a clear tendency to measurements being below the regression line. This is probably due to the measurements being below background in the region and a horizontal regression line would be more appropriate, suggesting a piecewise regression model. This is an important observation as the usual convolution models used in the de-convolution of signals from measurement in array data does not take an IDL into account. This will, however, lead to more complicated IDL and background correction calculations and is left for future research. Over-dispersion of gene count data from NGS data is well documented see e.g [17,18]. In this paper we resolved this by using the *quadratic mean variance negative binomial model*, i.e. NB2, when detecting differentially expressed genes, whereas we used the *linear meanvariance negative binomial model*, i.e. NB1, when analysing the IDL. NB1 is not supported in the edgeR package but we discovered that NB1 was sufficient for the negative binomial regression by residual plots (Additional file 6, Figures C & D, and E & F) and comparison of deviances. We found that resolving the problem of finding the most appropriate dispersion model estimates for NGS data was outside the scope of the present paper, but it is an important topic for future research.

The ability to detect single genes by exon microarray and tag-seq was exemplified by analysing the mRNA expression levels as a function of sample purity for three different B-cell mRNA transcripts. The mRNA expression levels of *CD74*, *HLADRA*, and *BCL6* followed a linear relationship as a function of sample purity for exon microarray, whereas larger fluctuations were observed for tag-seq (Figure 3). Feng *et al*. demonstrated an increased coefficient of variation when detecting low-abundant mRNA transcripts by tag-seq [15]. With increased sequencing depth, detection of low-abundant mRNA transcript will probably become more accurate [14]. Feng *et al*. described the relationship between total sequence volume and information of mRNA expression levels as a sigmoid function. This means the deeper the sequencing, the greater the information [15]. The present work demonstrates that the difference in the ability to detect the three genes above depends on the sample purity and the initial abundance in the pure B-cell sample. Tag-seq showed a tendency to have higher ability to detect highly abundant transcripts compared to exon microarray, supporting previous observations [5,14].

Based on the analysis of the three selected single genes, we demonstrated that the best detection ability corresponds to the highly abundant malignant B-cell transcripts, and there was an actual loss in the ability to detect low-abundant mRNA transcripts in samples with low purity. For *BCL6*, as a low-abundant gene, it was evident that a malignant B-cell frequency above 20-50% was required for reliable transcript detection. This supports the observed proportional relationship between the ability to detect genes and increasing sample purity.

The next obvious step for a follow-up study within this theme is to make serial dilutions of malignant B-cells into normal B-cells, T-cells and macrophages. Our results show that basic requirements of detection abilities can be identified and detection capabilities can be studied in a formal framework. Other directions for future studies include RNA-Seq on the entire poly-A fraction. This may help to detect even very low-abundant transcript, possibly helping to identify malignant B-cell specific transcript, i.e. alternative splicing isoforms, which are missed by tag-seq or more interestingly the fusion transcripts, which are cancer-specific and may dramatically help to create the conditions for establishing a future model system.

## Conclusions

In the present study, it was demonstrated that the developed methodological tools are valuable in assessing the ability to detect genes by high-throughput platforms capable of screening samples for tumour-specific mRNA transcripts, but more extensive studies are needed to give general advice on the choice of optimal technology. An important future perspective when assessing and comparing the detection capabilities of high-throughput platforms is to validate the usefulness of the proposed model system and analysis setup, by diluting pure malignant lymphoma samples with normal lymph node tissue.

## Methods

### Laboratory work

#### Samples of cell lines

The cell line, OCI-Ly8, provided by Dr. Hans Messer and Dr. Andreas Rosenwald was established at Ontario Cancer Institute (OCI). OCI-Ly8 was established from a patient with diffuse large B-cell lymphoma [19]. This cell line was maintained in RPMI1640 medium (GIBCO Invitrogen, NY, USA) including 10% foetal bovine serum (FBS, GIBCO Invitrogen, NY, USA). Cell line HEK293 (ATCC) was generated by transformation of normal human embryonic kidney cells with sheared adeonivrus DNA [20]. This cell line was maintained in Eagle’s Minimum Essential Medium (GIBCO Invitrogen, NY, USA) supplemented with 10% FBS. Both cell lines were cultured at 37°C in a CO_2_-incubator (BINDER, NY, USA) in a humidified atmosphere supplemented with 5% CO_2_. Cells were harvested in the exponential growth phase and sorted by a BD FACSAria II Cell Sorter (BD Bioscience, San Jose, CA, USA) to obtain eight cell populations, diluting OCI-Ly8 into HEK2932 in the cellular concentrations 0%, 0.5%, 1%, 5%, 10%, 20%, 30%, and 100% of OCI-Ly8 and subsequently stored in Trizol Reagent (GIBCOBRL Invitrogen, NY, USA). Cell line identity was analysed using 0.2 ng/μl extracted RNA from the cell lines as template in a multiplex PCR using the AmpFISTR Identified PCR amplification kit (Applied Biosystems, CA, USA). A fragment analysis of the amplified PCR product was performed by capillary electrophoresis by Eurofins Medigenomix GmbH, Applied Genetics, Germany. The resulting FSA files were analysed using the Osiris software (http://www.ncbi.nlm.nih.gov/projects/SNP/osiris/) given the following STR profiles: OCI-Ly-8: D5S818-4 (10, 13), D13S317-2 (11, 11), D7S820-1 (11, 12), D16S539-2 (9, 11), VWA-3 (17, 19), TH01-2 (6, 9.3), AMEL-4 (X, X), TPOX-3 (9, 11), CSF1PO-1 (11, 12), and HEK293: D5S818-4 (8, 9), D13S317-2 (12, 12), D7S820-1 (11, 11), D16S539-2 (9, 13), VWA-3 (16, 19), TH01-2 (7, 9.3), AMEL-4 (X, X), TPOX-3 (11, 11), CSF1PO-1 (11, 12).

#### Total RNA extraction

Total RNA was extracted from the eight cell populations using the Trizol Reagent and purified using the *mir*Vana miRNA Isolation Kit (AMBION INC., NY, USA) according to the manufacturer’s protocol. The quantity of extracted total RNA was measured on a NanoDrop ND-1000 spectrophotometer (Thermo Scientific, Denmark), and the quality of the total RNA was assessed using the Agilent RNA 6000 Nano kit and Nano Series II assay in combination with the Agilent 2100 Bioanalyzer (RIN values >9, Agilent Technologies, Denmark). Total RNA was stored at −80°C until use.

#### Affymetrix exon array

Exon microarray data were generated using 100 ng of total RNA. Ambion WT Expression Kit (AMBION INC, NY, USA) generated amplified and biotylated sense-strand cDNA from the total RNA according to the manufacturer’s protocol. Afterwards, single-strand cDNA was fragmented by UDG (uracil glycosylase) and APE1 (apurinic/apyrimidinic endonuclease) and biotin-labelled using the GeneChip Terminal Labeling and hybridisation Kit (Affymetrix, CA, USA) according to the manufacturer’s protocol. 5 μg of biotin-labelled cDNA were incubated with the Affymetrix GeneChip Human Exon 1.0 ST array at 45°C for 17 hours for hybridisation. Following hybridisation, non-specific bound material was removed by washing with the GeneChip Hybridisation, Wash and Stain kit (Affymetrix, CA, USA) and the GeneChip Fluidics Station 450 (Affymetrix, CA, USA). Detection of bound material was performed by scanning the array using the GeneChip Scanner 7G (Affymetrix, CA, USA).

#### Tag preparation and sequencing

For tag preparation, 2 μg of total RNA were incubated with magnetic Oligo-(dT) beads to capture the polyadenylated RNA fraction. Bound to the beads, total RNA was reverse transcribed into double-stranded cDNA using the SuperScript II kit (Invitrogen, NY, USA). The double-stranded cDNA was digested with the restriction enzyme *NlaIII* (New England Biolabs, MA, USA) to generate cDNA fragments from the most 3′-end CATG to the poly(A) tail. cDNA without the *NlaIII* recognition site was removed by washing. A biotinylated adaptor containing the recognition site for the restriction enzyme *MmeI* was linked to the 5′-end of the digested double stranded cDNA. Next, tags of 17 bp of double stranded cDNA were released from the Oligo-(dT)_25_ beads by *MmeI* (New England Biolabs, MA, USA). Tags were purified from a polyacrylamide gel by phenol-chloroform extraction and bound to Streptavindin beads. A second adapter containing an identification key of 3 bp unique for each sample was ligated to the tags. The adaptor-ligated tags from all samples were pooled and amplified by PCR. The PCR products were purified by excision from a 6% polyacrylamide gel (Invitrogen, NY, USA), followed by elution, ethanol precipitation and re-suspension (Illumina, MA, USA). The amount of adaptor-ligated tags was quantified using the NanoDrop ND-1000 spectrophotometer (Thermo Scientific, Denmark). The sample was stored at −20°C until sequencing. The sequencing procedure was carried out according to the manufacturer’s protocol. Adaptor-ligated tags from all samples were sequenced in an individual lane of a flow cell by the Solexa/Illumina Whole Genome Sequencer 1G analyser. Clustering and sequencing were performed with reagents from the cluster generation kit and sequencing kit (Illumina, MA, USA).

#### RT-qPCR

RT-qPCR data were generated using total RNA reverse transcribed into cDNA by the SuperScript III First-Strand Synthesis Supermix (Invitrogen, NY, USA ) and TaqMan reagents and assays (Applied Biosystems, CA, USA) according to the manufacturer’s protocol. 500 ng total RNA was mixed with 50 μM Oligo(dT) primer, 50 ng/μl random hexamer primer, and Annealing buffer. The sample was incubated in the thermal cycler at 65°C for 5 minutes to unfold loops together with secondary structures. Following incubation, 2x First-Strand Reaction mix and SuperScript III/RNaseOUT Enzyme mix were added to the sample, and the sample was incubated in the thermal cycler at 25°C for 5 minutes, 50°C for 50 minutes, and then 80°C for 5 minutes. The cDNA was used directly in RT-qPCR or stored at −20°C.

For RT-qPCR, 2x TaqMan Universal Master mix without AmpErase UNG (Applied Biosystems, CA, USA) and 20x TaqMan gene expression assay was mixed. The cDNA product was diluted 10 times, and 4 μl of the cDNA product was used as template. The cDNA product was amplified using a two-step thermal profile: 95°C for 10 minutes, followed by 50 cycles at 95°C for 15 seconds and 60°C for 1 minute.

The RT-qPCR was performed using cDNA product and the TaqMan gene expression assays (Applied biosystems, CA, USA) specific for *CD74* (Hs00959496_m1), *HLA*-*DRA* (Hs00219576_m1), and *BCL6* (Hs00153368). To correct for sample-to-sample variations in efficiencies and errors in quantification, the TaqMan Pre-Developed endogeneous control assay (Applied Biosystems, CA, USA), *TBP* (333769F), were used for normalisation. Amplified mRNA was detected by the Mx3000P QPCR instrument (Stratagene, CA, USA).

### Data processing and analysis

#### Generation of .CEL-files and gene summarization

CEL-files were generated by the Affymetrix GeneChip Command Console Software (AGCC) and deposited at the NCBI Gene Expression Omnibus (GEO) under accession number GSE40311. The .CEL-files were imported into Bioconductor using the aroma.affymetrix package. The .CEL-files were background corrected and quantile normalised by the RMABackgroundCorrection and QuantileNormalization functions from the aroma.affymetrix package [21]. Core probe intensities were summarised to gene expression levels by means of the ExonRMAPlm(, mergeGroups=TRUE) function using the customised cdf-file for core transcripts downloaded from http://bcgc.lbl.gov/cdfFiles/HuEx-1_0-st-v2,A20071112,EP/HuEx-1_0-st-v2,coreR3,A20071112,EP.cdf.

#### Extraction, annotation and gene summarization of tag sequences

The Illumina Pipeline software version 1.3 was used for image analysis and base calling, omitting chastity filtering, otherwise using default settings. Data were imported into the CLC Genomics Workbench 4.5 and sequence error corrected by the SAGEScreen method. The first 17 bp of the tag sequences were extracted from the output file according to the second adaptor containing the identification key for each sample. The unique tags were sorted and counted for each sample using the CLC Genomics Workbench 4.5 and exported to .txt-files. These text files were imported into Bioconductor. Due to a large library-to-library variation in the amount of obtained tags the set of tags in the libraries were randomly sub-sampled without replacement to equal the size of the smallest library.

Each unique tag sequence was annotated by mapping the reads with the anchoring NlaIII restriction site to the ENSEMBL Homo Sapiens cDNA reference transcriptome (release 63) downloaded from ftp://ftp.ensemble.org on both the coding and non-coding strands using Bowtie version 0.12.7 [22]. The setting –v1 was used allowing Bowtie to accept a maximum of one mismatch. ENSEMBL identifiers were via Entrez identifiers, mapped to gene symbols by use of the R-package Biomart and the org.hs.eg.db database. The total expression for each gene was calculated by summing all tags mapped to the same gene. Tag-seq data were deposited at GEO under accession number GSE40311.

### Statistical analysis

All statistical analyses were done with R Version 2.13.2 (64 bit Windows version) and a number of Bioconductor packages [23] as described below. Detailed session information is contained in Additional file 7. *P*-values were false discovery rate (FDR) corrected by the Benjamini-Hochberg procedure and 0.05 was used as significance level.

### Differentially expressed genes

#### Exon microarray

Since no replicates were present for the pure cell lines, it was assumed for exon microarrays that the expression value, *Y*_*gi*_, for the *g*’th gene out of *G* genes and the *i*’th sample out of two samples is *N*(*μ*_*gi*_, *σ*^2^) - distributed. Then, under the null hypothesis of no difference in gene expression of gene g between sample 1 and 2, *Y*_*g*1_ − *Y*_*g*2_, is *N*(0, 2*σ*^2^) -distributed. If

$$s=\frac{\sqrt{\frac{1}{G-1}\sum {\left(Y_{g1}-Y_{g2}\right)}^{2}}}{2}$$

this means that *P*-values of the z-scores *z*_*g*_ = (*Y*_*g*1_ − *Y*_*g*2_)/*s* can be used to test the hypothesis of no difference in gene expression levels. The *P*-values of the z-scores can be FDR corrected by the Benjamini-Hochberg procedure as implemented in the Bioconductor package fdrtool.

#### Tag-seq

The Bioconductor package edgeR is designed for differential expression analysis of count data and is an implementation of the methods developed by Robinson and Smyth [24]. As recommended, we only keep tags that are sufficiently expressed in at least one of the samples by filtering tags where the count is not above 15 in any of the samples. The edgeR package models the counts, *Y*_*gi*_, of a gene *g* in sample *i* as a quadratic mean-variance negative binomial distributed random variable

$$Y_{\mathrm{gi}}∼NB2\left(M_{i}p_{\mathrm{gj}},\phi_{g}\right)$$

where Mi is the library size, pgi is the relative abundance of gene g, and ϕg is the dispersion parameter. When the negative binominal distribution was fitted, a common dispersion for all tags was estimated using the Cox-Reid profile-adjusted likelihood method. FDR corrected *P*-values for differentially expressed genes were determined using an exact test.

#### Detection above background

In order to determine genes detected above background, the DABG algorithm of Affymetrix Power Tools (APT) [25] version 1.14.3.1 with the instruction “apt-probeset-summarize –a dabg” was used to find *P*-values for the hypothesis that each exon belongs to the background. The .PGF and .CLF files were used for the exon array, and for the background correction, the antigenomic background probes were used. Summarising to gene level was performed as follows [26]: For each gene in each sample, the number of present exons was counted – presence is assumed whenever the *P*-value was below 0.005. If half or more than half of the exons were present, the gene was assumed to be present in that sample, or otherwise it was absent.

#### Hierarchical clustering

A hierarchical cluster analysis was performed on the differentially expressed genes. The hierarchical cluster analysis was based on the R-function hclust using complete linkage as the agglomeration method and Eucledian distance as dissimilarity measure.

#### Instrumental detection limit and method detection limit

The instrumental detection limit (IDL) is defined as the analyte concentration required to produce a signal that is distinguishable from the blank level with a particular statistical confidence [27]. Formally, the blank level is assumed equal to *b*, the random variable *Y*(*t*) denote a measurement of the analyte at concentration *t* ≥ 0 and the statistical confidence is chosen to be 95%. Then, the IDL is defined as

$$IDL=min\left\{t\geq 0:P\left(y\left(t\right)>b\right)\geq 0.95\right\}\text{.}$$

#### Exon microarray measurement model

For exon microarray, the concentration, *t*, should be understood as the light intensity arising from a particular mRNA transcript hybridised to the array. It is normally assumed that this light-intensity is directly proportional to the number of mRNA transcripts in the sample. The coefficient of proportionality depends on e.g. the array type and reaction efficiency. Hence, for exon microarrays, the following measurement model is assumed

$$Y\left(t\right)=t+b+\varepsilon \left(t\right)\text{,}$$

where *ε*(*t*) is assumed to be *N*(0, *σ*^2^) -distributed. In this case

$$\begin{array}{l} IDL=min\left\{t\geq 0:P\left(t+b+\varepsilon \left(t\right)>b\right)\geq 0.95\right\}\\ \;=min\left\{t\geq 0:P\left(\varepsilon \left(t\right)>-T\right)\geq 0.95\right\}\\ \;=1.64\sigma \text{.} \end{array}$$

The method detection limit (MDL) will in practice depend on the sample purity. If *p*, 0 ≤ *p* ≤ 1, is denoted as the ratio between the tumour and normal samples, the following measurement model is assumed

$$Y\left(t\right)=pt+b+\varepsilon \left(t\right)\text{.}$$

Then, it is straightforward to calculate the MDL as a function of the sample purity *p* in the following way

$$\begin{array}{l} MDL\left(p\right)=\text{min}\left\{t\geq 0:P\left(pt+b+\varepsilon \left(t\right)>b\right)\geq 0.95\right\}\\ \;=min\left\{t\geq 0:P\left(\frac{\varepsilon \left(t\right)}{p}>-t\right)\geq 0.95\right\}\\ \;=\frac{1.64\sigma }{p}\\ \;=\frac{IDL}{p}\text{.} \end{array}$$

This shows that the MDL is inversely proportional to the sample purity. The IDL can be estimated by dilution curves as follows: Let the gene expression be measured at the following dilutions of the *k*’th transcript, *t*_*k*_*x*_*i*_ where 0 = *x*_1_ ≤ ⋯ ≤ *x*_*n*_ = 1, then, for exon microarray, this means that *b*, *t*_*k*_ and *σ* can be estimated by the linear regression model

$$y_{\mathrm{kt}}=t_{k}x_{t}+b+\varepsilon_{\mathrm{kt}}$$

where *ε*_*ki*_ ~ *N*(0, *σ*^2^). This is done by means of least squares regression as implemented in the lm-function in R. Model control is performed by plotting the standardised residuals vs. the fitted values and normal probability plots of the standardised residuals. Standardised residuals above 3 are considered outliers and plotted on a log-transformed scale.

#### Tag-seq measurement model

For tag-seq, concentration, *t*, should be understood as the expected count number of a particular mRNA transcript. It is normally assumed that the expected count number is directly proportional to the number of mRNA transcripts in the sample. The coefficient of proportionality depends on e.g. the sequencing method and coverage depth. Hence, for tag-seq, it is assumed that *b* = 0. In the following we calculate the IDL for two models incorporating over-dispersion.

First consider the linear mean-variance negative binomial distribution NB1 where *Y*(*t*) is *NB*1(*μ*, *θ*) -distributed, i.e. *NB*1(*μ*, *θ*) is the linear mean-variance negative binomial distribution, with dispersion parameter *θ*, mean *μ* and variance *μ* + *θμ*. The pmf of this distribution is [28]

fk=Γk+μθΓμθΓk+1θ1+θk1+θ−μθ.

In this case

$$\begin{array}{l} IDL=min\left\{t\geq 0:P\left(Y\left(t\right)>0\right)\geq 0.95\right\}\\ \;=min\left\{t\geq 0:1-P\left(Y\left(t\right)=0\right)\geq 0.95\right\}\\ \;=min\left\{t\geq 0:{\left(1+\theta \right)}^{-\frac{t}{\theta }}\leq 0.05\right\}\\ \;=min\left\{t\geq 0:t\geq \frac{\theta \text{log}20}{\text{log}\left(1+\theta \right)}\right\}\\ \;=\frac{\theta }{\text{lo}{\text{g}}_{20}\left(1+\theta \right)}\text{.} \end{array}$$

This implies that in order to reliably observe an mRNA transcript, the expected count for the mRNA transcript should be more than *θ*/log_20_(1 + *θ*) counts in the specific experimental setup.

Next, consider the quadratic mean-variance negative binomial distribution NB2 where *Y*(*t*) is *NB*2(*t*, *θ*) -distributed, i.e. *NB*2(*μ*, *θ*) is the quadratic mean-variance negative binomial distribution, with dispersion parameter *θ*, mean *μ* and variance *μ* + *θμ*^2^. The pmf of this distribution is

fk=Γk+1θΓ1θΓk+1μθ1+μθk1+μθ−1θ.

In this case

$$\begin{array}{l} IDL=min\left\{t\geq 0:P\left(Y\left(t\right)>0\right)\geq 0.95\right\}\\ \;=min\left\{t\geq 0:1-P\left(Y\left(t\right)=0\right)\geq 0.95\right\}\\ \;=min\left\{t\geq 0:{\left(1+t\theta \right)}^{-\frac{1}{\theta }}\leq 0.05\right\}\\ \;=min\left\{t\geq 0:t\geq \frac{20^{\theta }-1}{\theta }\right\}\\ \;=\frac{20^{\theta }-1}{\theta }\text{.} \end{array}$$

This implies that in order to reliably observe an mRNA transcript, the expected count for the mRNA transcript should be more than (20^*θ*^ − 1)/*θ* counts in the specific experimental setup.

The method limit of detection (MLD) for both the NB1 and NB2 model will in practice depend on the sample purity. If *p*, 0 ≤ *p* ≤ 1, is defined to be the ratio between the amount of tumour and normal sample, the following measurement model is assumed

$$Y\left(t\right)∼NBx\left(pt,\theta \right),where\;x=1,2\text{.}$$

Then, it is straightforward to calculate the MDL as function of *p*

$$MDL\left(p\right)=\frac{IDL}{p}\text{.}$$

This shows that the MDL is inversely proportional to the sample purity. The IDL is estimated by dilution curves in the following way: Let the transcript be measured at the following dilutions of the k’th transcript, *t*_*k*_*x*_*i*_, where 0 = *x*_1_ ≤ ⋯ ≤ *x*_*n*_ ≤ 1, then, for tag-seq, this means that *t*_*k*_ is estimated by a negative binomial regression model with log-link

$$y_{\mathrm{kt}}∼\text{NBx}\left(\mu_{\mathrm{kt}},\theta \right)$$

where *a*_*k*_ = log *t*_*k*_ and *x* = 1, 2. This is done by means of maximum likelihood estimation of the parameters as implemented with the gamlss-function in R [29]. Model control can be performed by plotting the quantile residuals vs. the fitted values and normal probability plots of the quantile residuals. Models can be compared by the deviance.

#### Cross platform reproducibility analysis

We assessed the cross platform reproducibility by means of correspondence curves [30]. A correspondence curve is constructed by ranking the genes according to their significance for each of the two experiments. Then the number of genes in common is plotted against the number of significant genes. Under perfect concordance this plot will behave like *y* = *x* and under random sampling it will behave like *y*=*x*^*2*^. The acceptance region at a given level of significance for the hypothesis of random sampling can be constructed by the hypergeometric distribution [12].

#### RT-qPCR

For determination of mRNA expression levels by RT-qPCR measurements were performed in triplicates, and the average cycle thresholds (Cq) were used to determine fold-change. The mRNA expression levels were determined by the ∆∆Cq method [24] using *TBP* as reference gene and the pure OCI-Ly8 cell line as reference sample. No reverse transcription and no template control samples were used as controls.

## Competing interests

The authors declare they have no competing interests.

## Authors' contributions

MBK was responsible for the cell line culturing, flow sorting, sequencing, array analysis, RT-qPCR validation, data interpretation and drafted the manuscript. AEB implemented the tag-seq data analysis workflow, and MRD implemented the exon array data analysis workflow. AEB and MRD participated in data interpretation and paper drafting. KSB assisted with array analysis, AS with flow sorting, JSB with cell culturing and MS and SF assisted with data analysis. MN, HEJ, KLN, KD and MB conceived the study, and participated in its design and coordination and assisted in drafting the manuscript. All authors read and approved the final manuscript.

## Supplementary Material

Additional file 1 Analysis workflow. Click here for file

Additional file 2 Correspondence curve (solid blue line) and 5% acceptance region (grey area) for the test of independence in gene selection between the exon microarray and tag-seq platforms. Click here for file

Additional file 3 Differentially expressed mRNA transcripts between the pure OCI-Ly8 and HEK293 samples identified by tag-seq. Click here for file

Additional file 4 Differentially expressed mRNA transcripts between the pure OCI-Ly8 and HEK293 samples identified by tag-seq. Click here for file

Additional file 5 Hierarchical clustering after subsampling of all cell populations based on all genes (A) exon microarray and (B) tag-seq. Click here for file

Additional file 6 Model control for the linear, NB2 and NB1 models. Click here for file

Additional file 7 Session information. Click here for file
