# Supplementary figures and images for: A model system for assessing and comparing the ability of exon microarray and tag sequencing to detect genes specific for malignant B-cells
Source: BMC Genomics. 2012 Nov 5;13:596. doi: 10.1186/1471-2164-13-596 (PMC3505742; doi:10.1186/1471-2164-13-596)

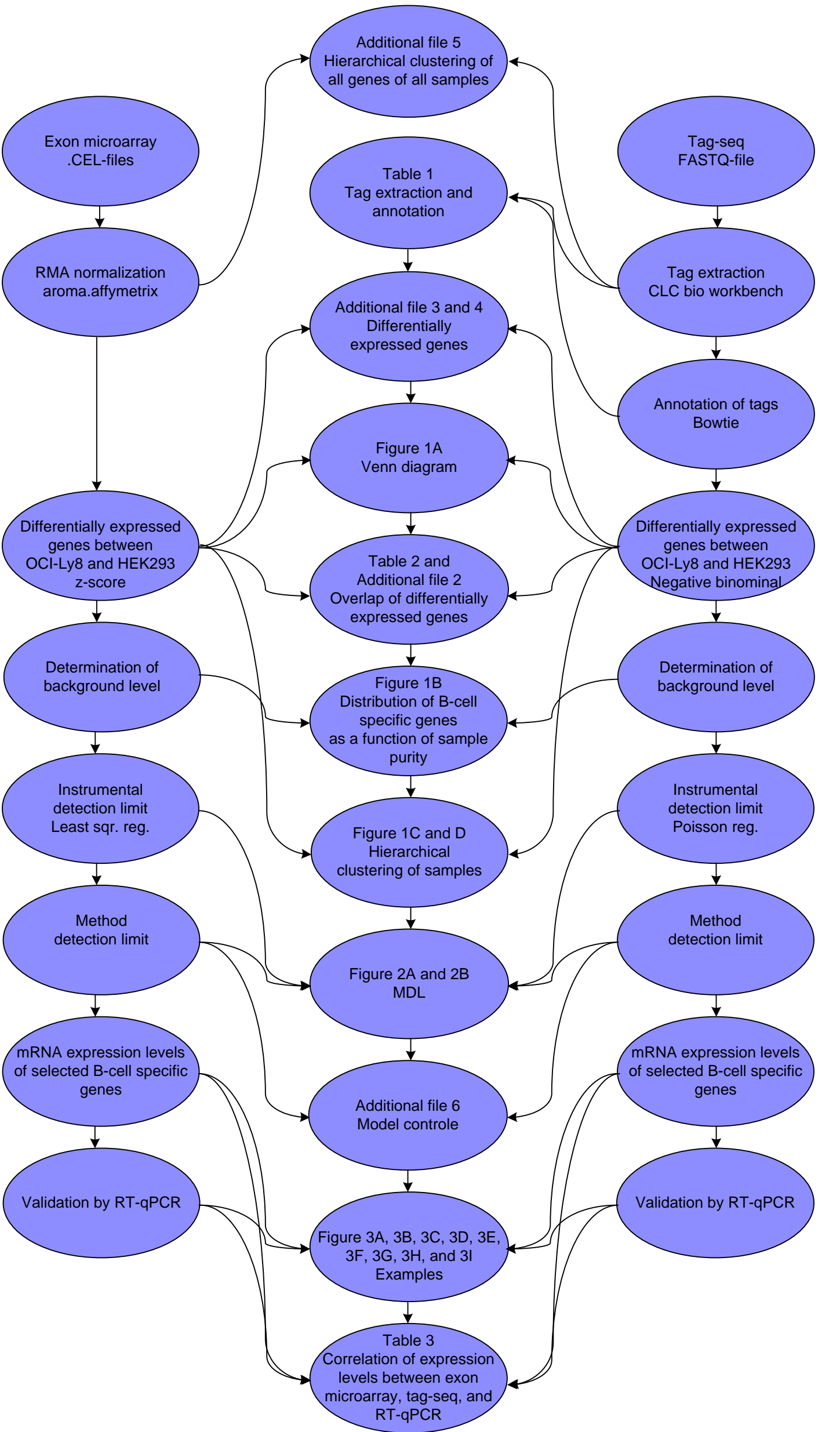

Supplement: Additional file 1 — Analysis workflow. [file 1471-2164-13-596-S1.pdf]

# Correspondence Curve

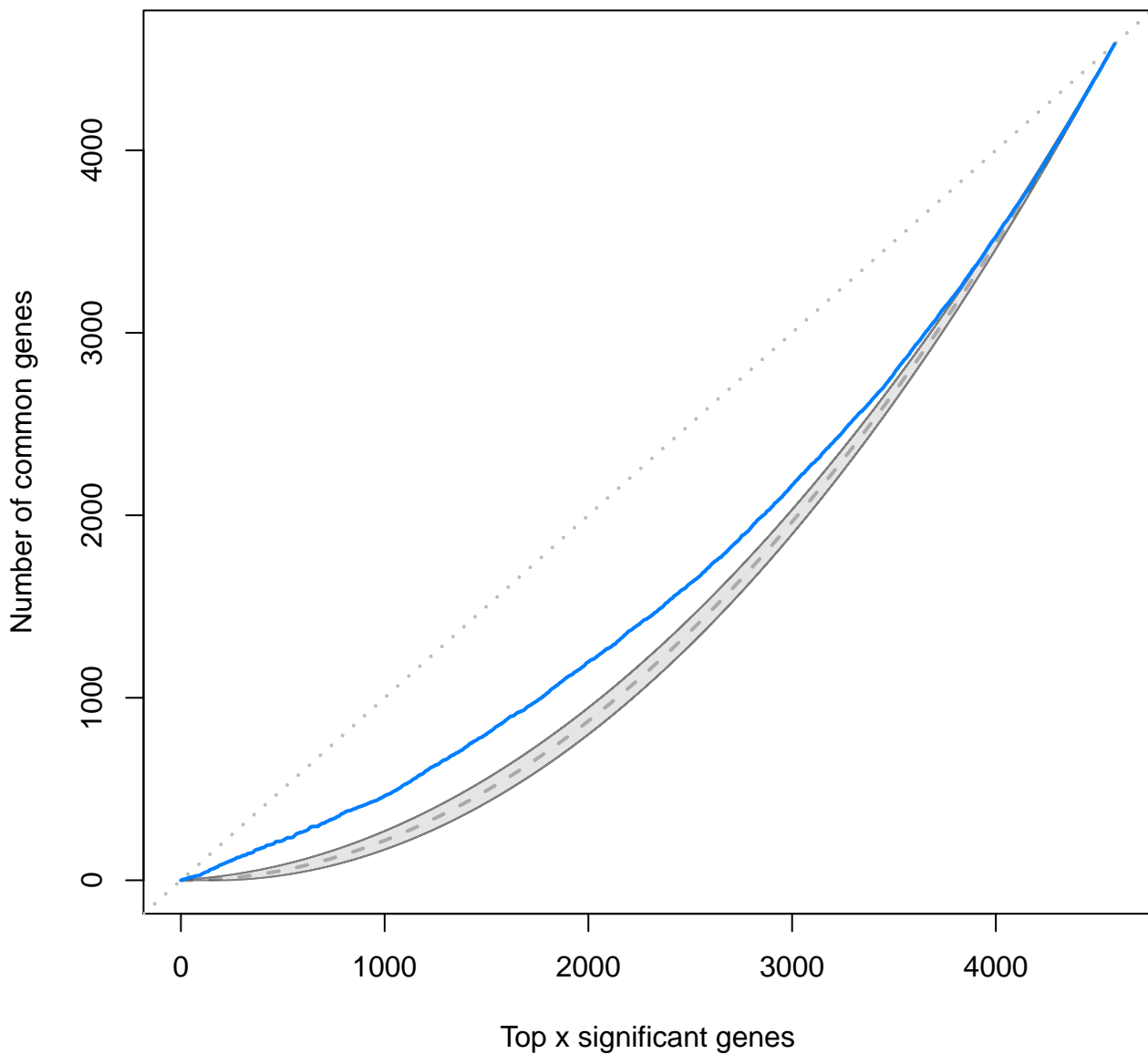

Supplement: Additional file 2 — Correspondence curve (solid blue line) and 5% acceptance region (grey area) for the test of independence in gene selection between the exon microarray and tag-seq platforms. [file 1471-2164-13-596-S2.pdf]

**A****Exon (all data)**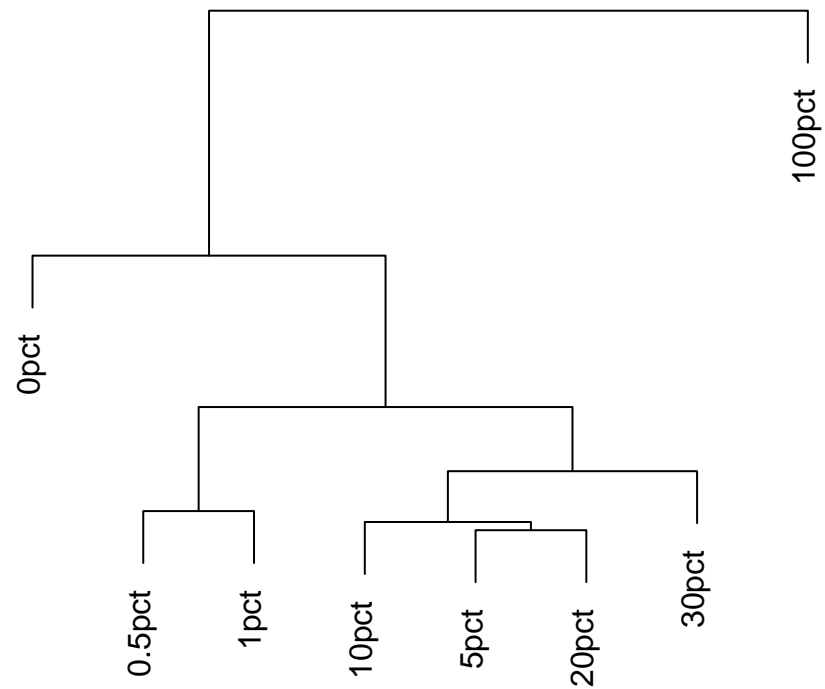**B****Tag-seq (all data)**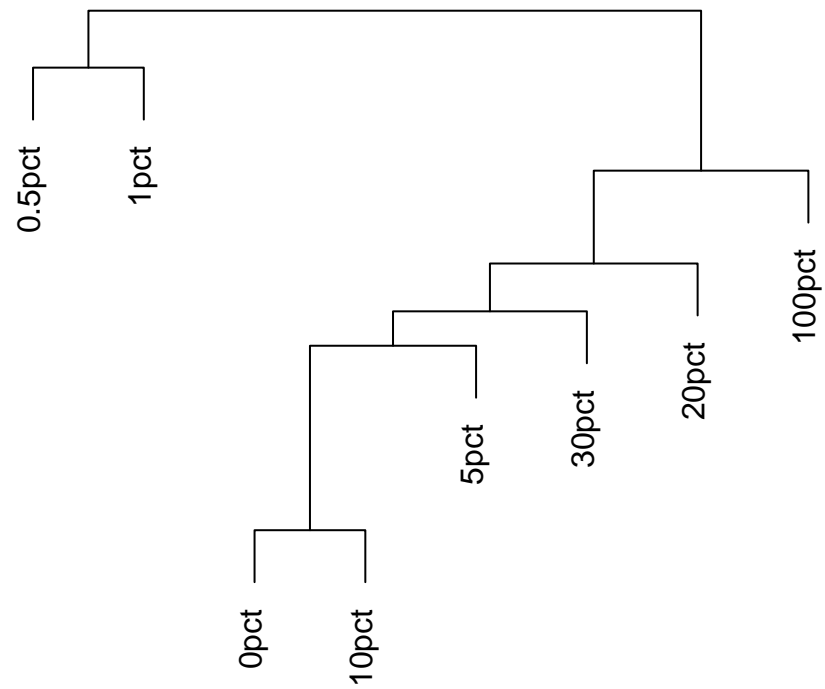

Supplement: Additional file 5 — Hierarchical clustering after subsampling of all cell populations based on all genes (A) exon microarray and (B) tag-seq. [file 1471-2164-13-596-S5.pdf]

**A**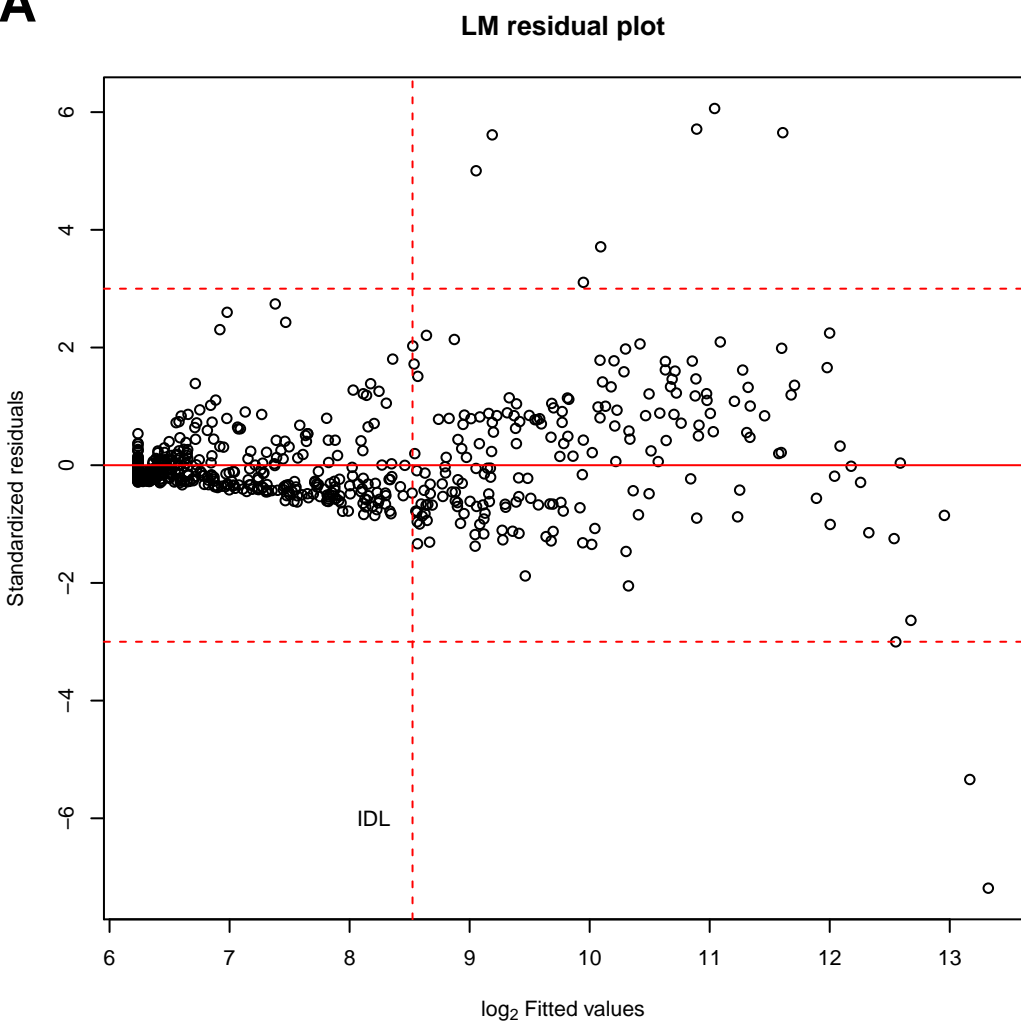**B**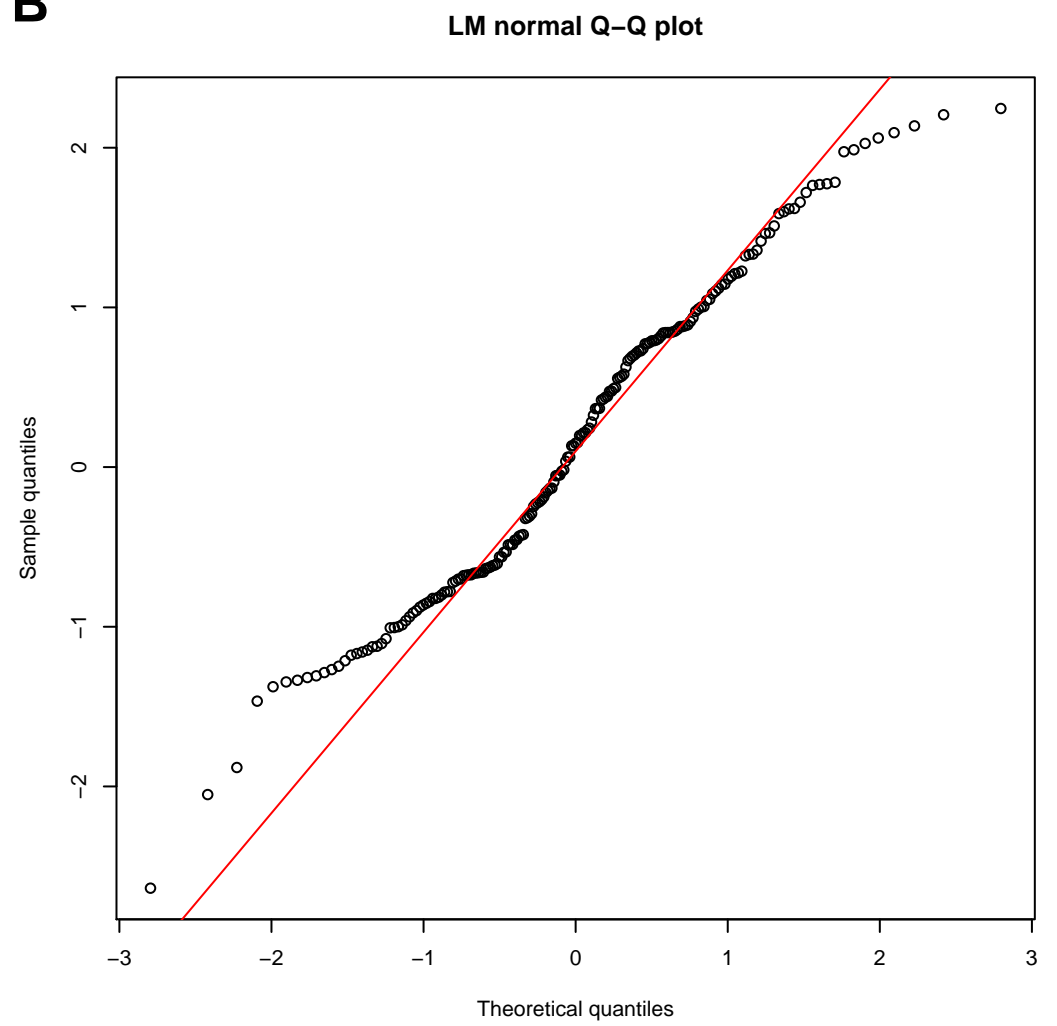**C**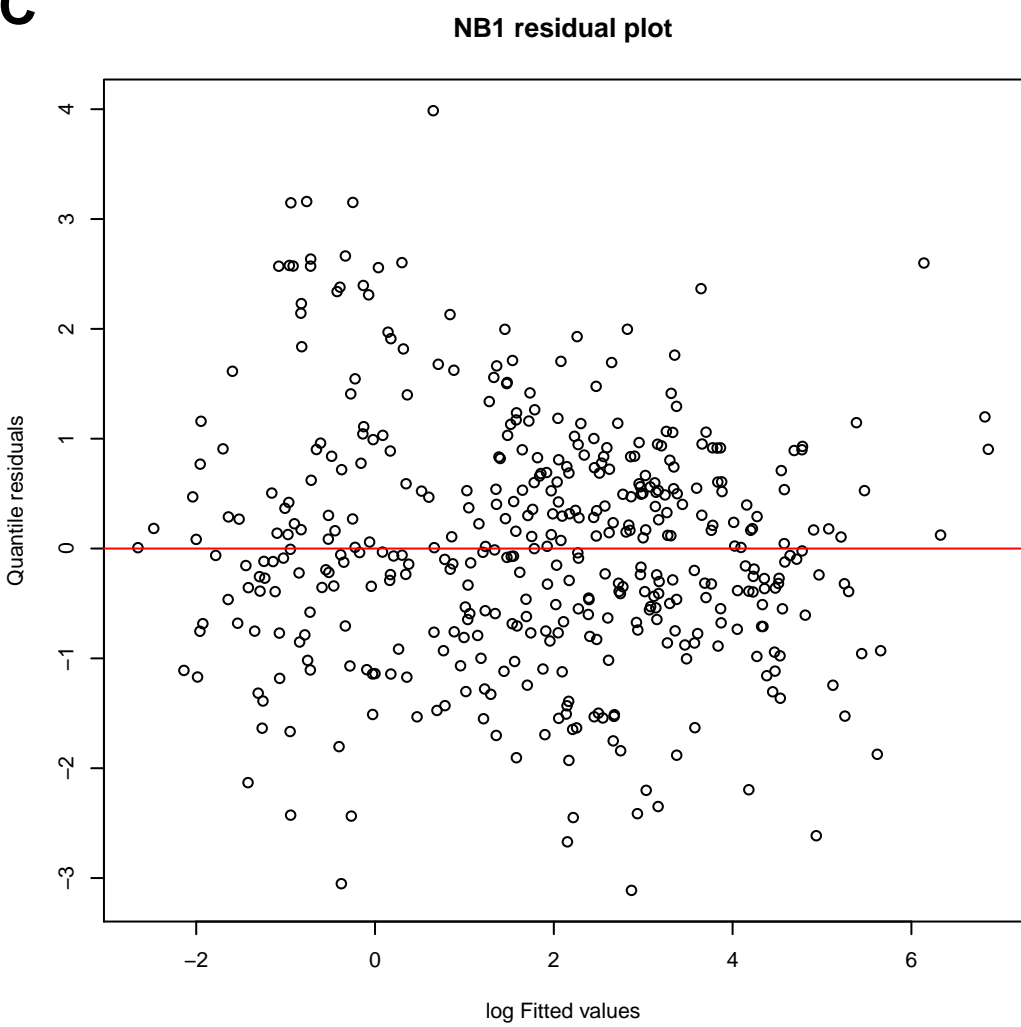**D**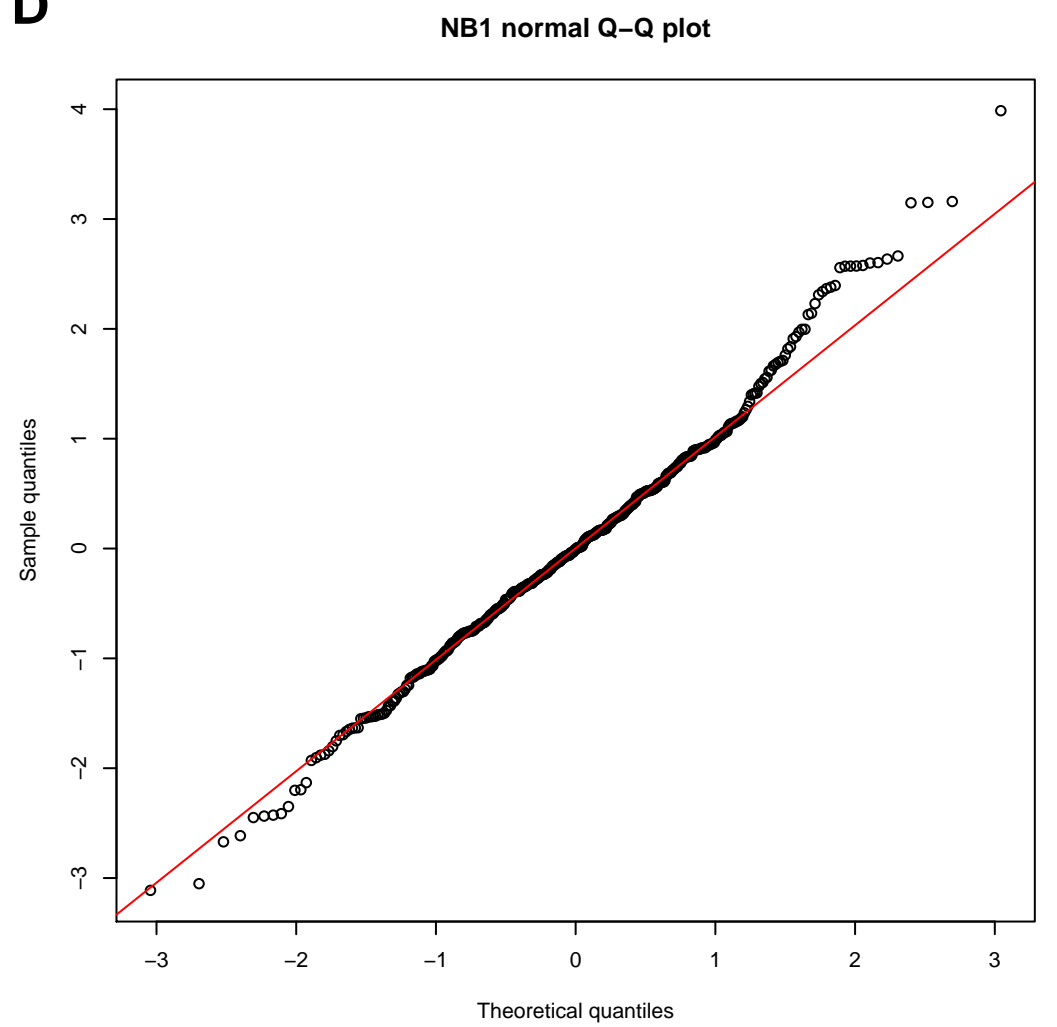**E**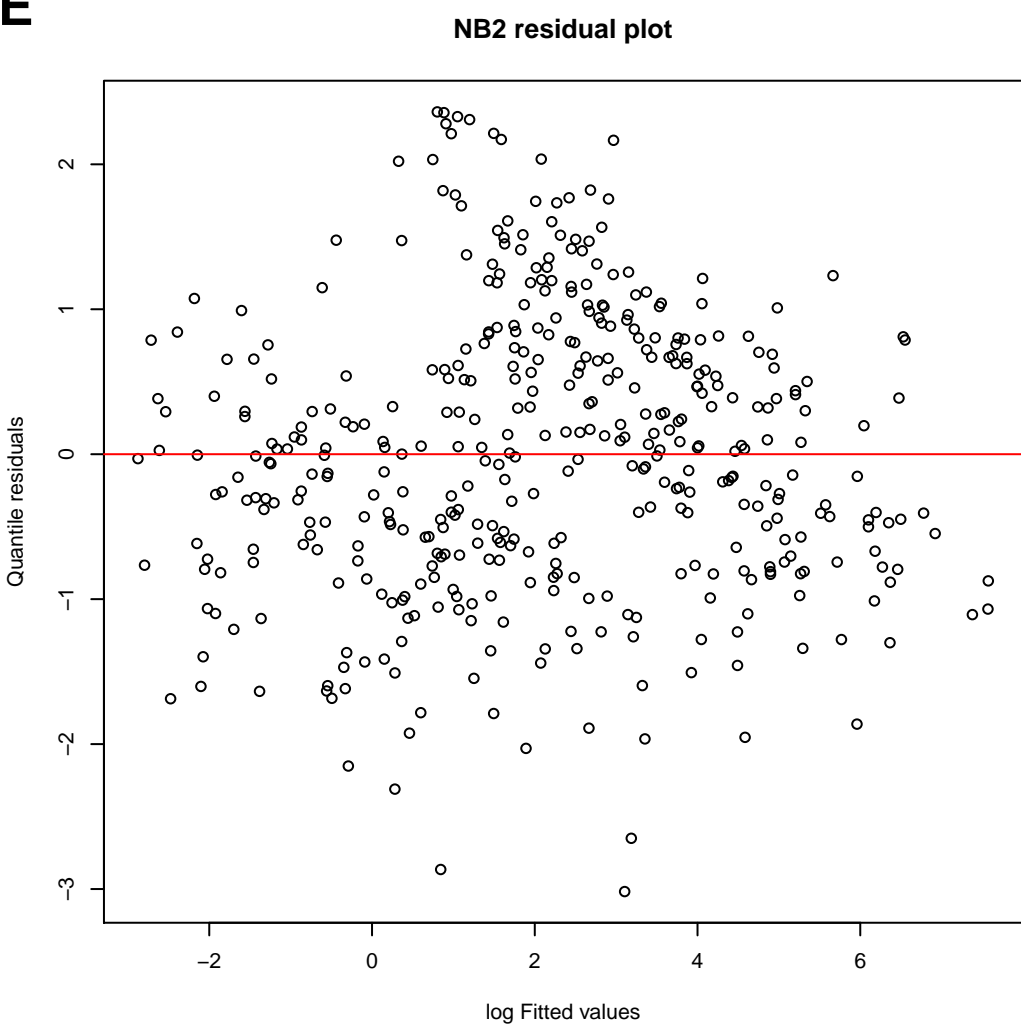**F**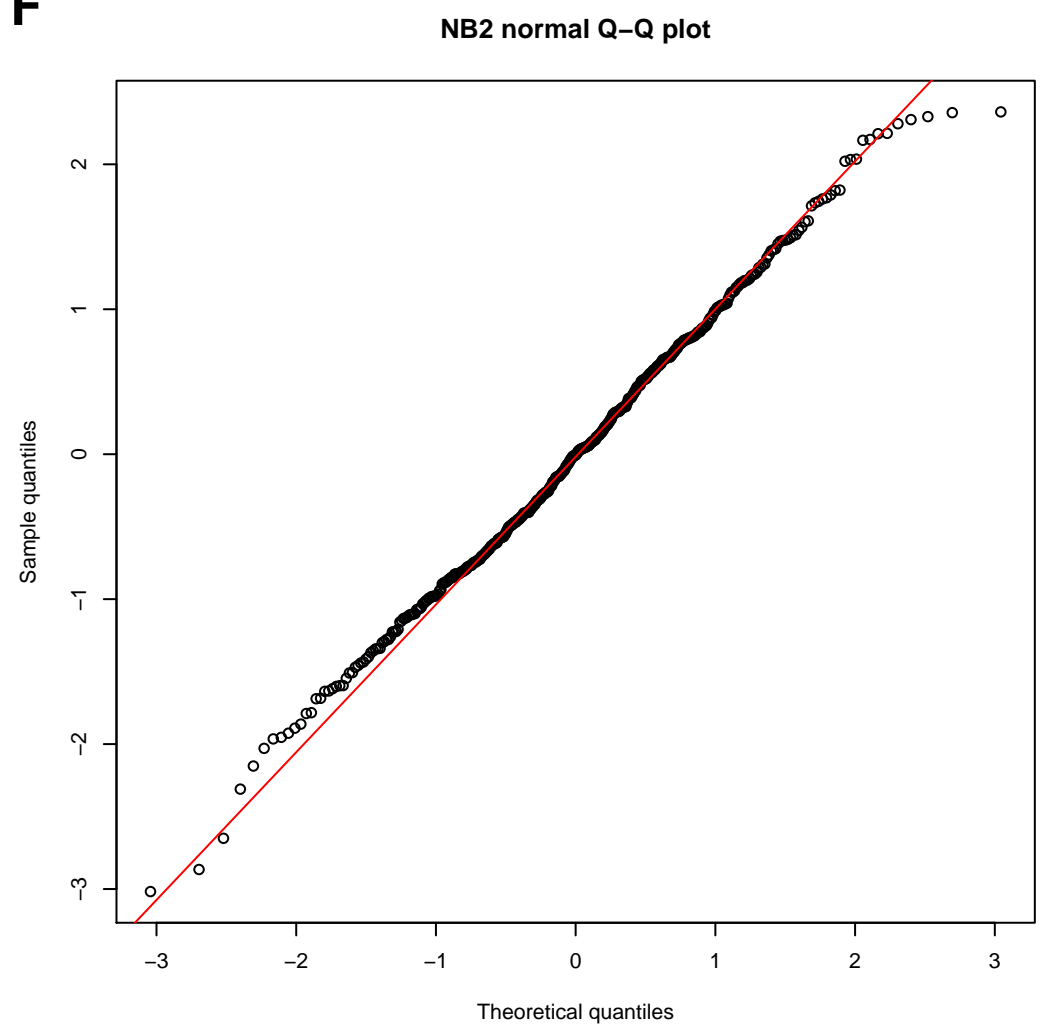

Supplement: Additional file 6 — Model control for the linear, NB2 and NB1 models. [file 1471-2164-13-596-S6.pdf]
